# Diagnosis and biomarkers of predementia in Alzheimer's disease

**DOI:** 10.1186/1741-7015-8-89

**Published:** 2010-12-22

**Authors:** Orestes V Forlenza, Breno S Diniz, Wagner F Gattaz

**Affiliations:** 1 Laboratory of Neuroscience (LIM 27), Department and Institute of Psychiatry, Faculty of Medicine, University of São Paulo, São Paulo, Brazil

## Abstract

In view of the growing prevalence of Alzheimer's disease (AD) worldwide, there is an urgent need for the development of better diagnostic tools and more effective therapeutic interventions. At the earliest stages of AD, no significant cognitive or functional impairment is detected by conventional clinical methods. However, new technologies based on structural and functional neuroimaging, and on the biochemical analysis of cerebrospinal fluid (CSF) may reveal correlates of intracerebral pathology in individuals with mild, predementia symptoms. These putative correlates are commonly referred to as AD-related biomarkers. The relevance of the early diagnosis of AD relies on the hypothesis that pharmacological interventions with disease-modifying compounds are likely to produce clinically relevant benefits if started early enough in the continuum towards dementia. Here we review the clinical characteristics of the prodromal and transitional states from normal cognitive ageing to dementia in AD. We further address recent developments in biomarker research to support the early diagnosis and prediction of dementia, and point out the challenges and perspectives for the translation of research data into clinical practice.

## Introduction

Alzheimer's disease (AD) is the most common dementing disorder in older people. As a consequence of population aging worldwide, a fourfold increase in the prevalence of AD is expected to occur over the next decades. Recent estimates foresee that more than 80 million individuals will be affected by the disease by 2040, which is a natural consequence of the age-dependent increase in the number of incident cases of AD [1-3]. An important contemporaneous challenge in the management of AD is to establish its early diagnosis, or, ideally, to identify the cases of AD prior to the actual onset of dementia. This requires the development of new diagnostic tools to predict the dementia outcome among older people with very mild symptoms of cognitive dysfunction, or even in asymptomatic individuals. Although a few promising methods have been experimentally validated, the translation of the current knowledge into clinical practice still requires methodological pruning and guidance by operational criteria.

The National Institute on Aging and the Alzheimer's Association have recently convened working groups to re-edit the diagnostic criteria for AD dementia, taking into account the vast expansion of the knowledge of the neurobiology of the disease, most of which was obviously unavailable by the time the original National Institute of Neurological and Communicative Disorders and Stroke-Alzheimer's Disease and Related Disorders Association (NINCDS-ADRDA) criteria were launched 26 years ago [4,5]. Another important accomplishment of these workgroups was to revise the clinical and biological correlates of AD in the symptomatic predementia phase, yielding the proposition of the diagnostic criteria for 'mild cognitive impairment (MCI) due to AD' [6]. The authors incorporated the use of biomarkers to define three levels of certainty of the clinical diagnosis, given the characterization of mild cognitive deficits in non-demented older people: (i) 'MCI of a neurodegenerative etiology', in the presence of the typical clinical presentation of individuals who are at an increased risk of progression to AD dementia, but have negative or ambiguous biomarker evidence of the underlying AD pathology; (ii) 'MCI of the Alzheimer type', when the subject meets the MCI criteria above and, in addition, has one or more topographic biomarkers associated with the downstream effects of the AD pathology (for example, MRI evidence of medial temporal lobe atrophy, or fluorodeoxyglucose positron emission tomography (FDG-PET) evidence of decreased temporomedial metabolism); and (iii) 'prodromal Alzheimer's dementia', when the subject meets the MCI criteria above and, in addition, has a positive biomarker for the molecular neuropathology of AD (such as molecular imaging of intracerebral amyloid with PET, or the typical pattern of the AD-related cerebrospinal fluid (CSF) biomarkers, as will be discussed below. The latter proposition does not require, but is reinforced by the topographic (downstream) evidence of the AD pathological process, as provided by structural or functional neuroimaging [6].

In this review article, we address the clinical characteristics of the prodromal stages of AD and the transitional states from normal cognitive ageing and dementia. We further present recent developments in biomarker research, and the perspectives of using these techniques to reinforce the clinical diagnosis of AD at predementia stages.

## Alzheimer's disease: translating neurobiological knowledge into clinical practice

**Figure 1:**
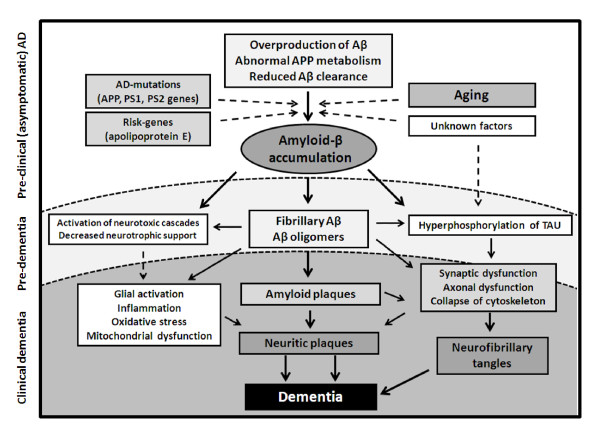
**Hypothetical model of the pathological processes in Alzheimer's disease (AD), focusing on the amyloid β peptide (Aβ) cascade**. (Other relevant mechanisms have been omitted or presented in a secondary perspective for didactic purposes.) Dotted arrows indicate possible or secondary mechanisms affecting core pathological processes within the amyloid cascade. Background shades of gray separated by dotted lines are a schematic representation to integrate the progression of pathological events along with the development of the cognitive syndrome of AD (these thresholds are arbitrary and not experimentally validated, and represent the authors' point of view of the disease process). Three clinical phases of the disease are defined: presymptomatic (or preclinical) AD may last for several years or decades until the overproduction and accumulation of Aβ in the brain reaches a critical level that triggers the amyloid cascade; in the predementia phase, compatible with the definition of mild cognitive impairment secondary to AD, early stage pathology is present in varying degrees, from mild neuronal dystrophy to early stage Braak pathology, according to individual resilience and brain reserve. Finally, in the clinically defined dementia phase, there is a progressive accumulation of the classical pathological hallmarks of AD (that is, neuritic plaques and neurofibrillary tangles), bearing relationship with the progression of cognitive deficits and the magnitude of functional impairment. APP = amyloid precursor protein; PS1/2 = presenilin 1/2; TAU = microtubule-associated protein Tau.

AD is a chronic neurodegenerative disease with well defined pathological markers, mostly affecting medial temporal lobe and associative neocortical structures. Neuritic plaques and neurofibrillary tangles, the pathological hallmarks of AD, are primarily related to the overproduction and aggregation of the amyloid β peptide (Aβ) within the brain, and to the hyperphosphorylation of Tau protein in affected neurons. These abnormalities lead to the activation of neurotoxic cascades and to cytoskeletal changes that eventually cause neuronal dysfunction and death. Neurofibrillary tangles appear first in allocortical structures, whereas amyloid plaques may first be found in the neocortex [7]. In addition to amyloid accumulation and neurofibrillary pathology, synaptic dysfunction leading to neuronal dystrophy are phenomena proxy to the structural changes of the brain, which ultimately triggers the clinical syndrome that characterizes incipient AD [8]. The cognitive manifestations associated with this process are compatible with subtle damage to hippocampal and related limbic and prefrontal structures, and may last for many years until the functional burden becomes severe enough to surmount the dementia threshold [9] (Figure 1).

There is evidence of a long preclinical phase in AD, in which the aforementioned abnormalities gradually accumulate in affected brain areas prior to the presentation of significant cognitive decline and dementia. Recent models based on neuropathological, biochemical and neuroimaging methods have proposed that intracerebral amyloidosis precedes the onset of cognitive symptoms by several years, if not decades. Autopsy studies have shown that intracerebral amyloidosis may be observed in some subjects as early as in the third or fourth decades of life, with increasing magnitude in late middle age, and highest estimates in old age [10-12]. The exact proportion of amyloid-positive normal adults who will ultimately develop AD is still uncertain, and critically dependent on the age and genetics of the cohort; yet, cortical amyloid load in cognitively normal older adults seems to be associated with a higher rate of progression to symptomatic AD in the long term [13].

It is a difficult task to clinically differentiate incipient AD from normal cognitive ageing and from the subtle cognitive changes that arise in other forms of dementia in the prodromal phases. In the early stages, patients with AD may present with mild but persistent (and often progressive) cognitive deficits, albeit not severe enough to warrant the diagnosis of dementia. In the recent literature, individuals in this predementia stage of AD have been most commonly categorized according to the definition of MCI [14]. However, it is widely accepted today that the cross-sectional diagnosis of MCI selects a clinically and biologically heterogeneous group of patients, which limits its prognostic value [15]. Given the insidiously progressive nature of most neurodegenerative illnesses, among which AD represents the most prevalent condition, it is reasonable to assume that most patients who are prone to become demented will present at early stages with symptoms compatible with the definition of MCI. Nevertheless, the reciprocal assumption may not be true, given the fact that many persons who fulfill diagnostic criteria for MCI at one particular assessment will not evolve to dementia at all.

**Figure 2:**
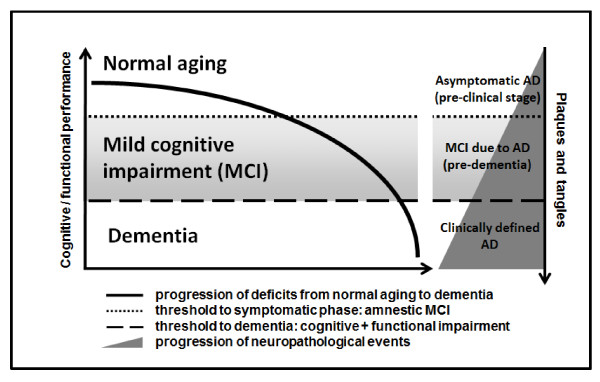
**Relationship between the progression of cognitive and functional symptoms and the neuropathological events in the transition from asymptomatic Alzheimer's disease (AD) to mild cognitive impairment due to AD and clinically manifest dementia of the AD type**.

Despite this, the long predementia phase in AD constitutes a unique time frame to search for clinical and neurobiological tools to reinforce the cross-sectional diagnosis and to predict the dementia outcome. The neuropathological features of subjects with amnestic MCI are intermediate between those found in cognitively normal and demented individuals (Figure 2). In a clinicopathological study, most patients with amnestic MCI did not meet the neuropathologic criteria for AD, but their pathological findings suggested a transitional state of evolving AD, given the involvement of medial temporal lobe structures likely accounting for the memory impairment [16].

Subtle changes related to the pathological process may be quantified in asymptomatic or mildly symptomatic patients by the assessment of humoral fluids, mostly cerebrospinal fluid (CSF), or by using advanced neuroimaging methods. Therefore, the rationale for the search for biological markers in AD is to increase diagnostic accuracy at early stages of the disease process. The correct use of this information may help identify subjects at risk of developing dementia upon follow-up. However, the clinical benefits will critically depend on the availability of more efficacious therapies to halt cognitive decline and, ideally, to prevent dementia. These include the development of more specific pharmacological interventions targeting the pathological cascades of AD.

## Predementia in Alzheimer's disease

The cognitive syndrome of early stage AD is primarily represented by encoding and retrieval deficits, resulting in episodic memory impairment and diminished capacity to learn new information [17-19]. Through clinical assessment, such deficits may be revealed by memory tests such as story/picture recall and word list learning. However, the specificity of these findings may be questionable, since similar deficits can be found in other prevalent conditions in older people, namely depression and cerebrovascular disease. Studies suggest that AD-prone patients fail to benefit from associative learning strategies and from mnemonic cues (for example, cued delayed recall paradigms). In the recent literature, short-term and long-term memory binding deficits have been suggested to be strong predictors of AD in older adults [19]. These subfunctions of the memory process refer to the ability to hold multiple sources of information in memory, and to bind together different aspects of one given stimulus in order to form integrated memories. These abilities are required for associative learning, and binding deficits may be early signs of hippocampal dysfunction. In fact, AD patients have difficulties in learning associations between two or more characteristics of the same object (for example, shape and color), distinct verbal contents of one given idea, the association between faces and names, and also to integrate spatial locations to other mnemonic contents. The clinical progression towards dementia includes the additional impairment of at least one more cognitive domain, which is normally represented by executive dysfunction [20]. Recent data indicate a positive strong correlation between the magnitude of executive and functional impairment [21].

The characterization of the cognitive syndrome that best predicts the AD outcome in non-demented patients has been the focus of extensive research in the past decades. However, the clinical picture of predementia AD overlaps with the cognitive changes that occur in normal ageing and other pathological processes. Frequency estimates of cognitive impairment in the older population depend critically on the definition that is adopted to yield the classification of subjects as normal or impaired. However, these different definitions not always agree with respect to the procedures that need to be adopted to rule in and out subtle cognitive deficits. In addition, the output obtained from the classification of individuals according to one given definition of cognitive impairment is highly dependent on the setting. Community samples are more heterogeneous regarding to the etiology of cognitive deficits, given the higher representation of symptoms attributed to medical and psychiatric causes. In contrast, the proportion of subjects with underlying AD pathology tends to higher in tertiary services and specialized memory clinics, where most attendees are actively seeking diagnosis and treatment for their symptoms. Therefore, the diagnosis of cognitive impairment in community samples may favor sensitivity in detriment of specificity; conversely, the positive predictive value of the diagnosis tends to be higher in patients attending memory clinics. A good illustration of this problem was published by Stephan *et al*. [22], who showed that the prevalence of cognitive impairment in community dwelling older adults using different definitions was as variable as 0.1% to 42%. The authors concluded that the classification of individuals as cognitively impaired or normal depends critically on the way criteria are defined and operationalized. Each classification captures a unique group of individuals, with little concordance and varying prognostic value. Thus, there is an urgent need for an agreed-upon standard case definition to use as a criterion standard.

## Mild cognitive impairment

Among the various definitions that have been proposed to ascertain the clinical signs and symptoms attributed to the earliest stages of dementia, the Mayo Clinic description of MCI, launched by the seminal works of Petersen and collaborators [14], is perhaps the most widely used term in the recent literature. Originally, this definition emphasized the presence of memory complaints, with objective demonstration of lower than expected performance on memory tests; there should be a global preservation of intellectual function and no evidence of functional impairment. A higher risk to progress to AD upon follow-up (approximately 10% per year) was attributed to subjects diagnosed as with MCI. A few years later, the definition of MCI was broadened to encompass deficits in other cognitive domains, such as language, attention and executive functions, and also to differentiate cases with association of deficits on one, two or more cognitive domains (that is, amnestic and non-amnestic, single-domain or multiple-domain MCI) [23,24]. Specific patterns of cognitive impairment would indicate a higher risk of distinct dementia outcomes.

Several other clinical and epidemiological investigations have also demonstrated that patients with MCI progress more often to AD or to other dementias than older adults without objective evidence of cognitive impairment. However, a substantial variation in the annual progression rates from MCI to AD is observed across studies, ranging from low estimates of 3% to very high estimates of 40% to 50% in samples defined according to the Mayo Clinic diagnostic criteria for MCI [25,26]. Several reasons have been pointed out to explain these discrepancies in conversion rates, particularly the magnitude of cognitive deficits at baseline (even though within definition limits) and the imprecise definition of functional impairment to differentiate MCI from dementia. Considering the arbitrary psychometric threshold for caseness based on the performance on cognitive tests (usually defined as 1.0 to 1.5 standard deviations below age-corrected and education-corrected population norms), the definition of MCI still accepts a relatively wide range of cognitive deficits, both in quantitative and qualitative terms. In addition, no guidelines have so far been provided to operationalize the cognitive assessment of patients (that is, which cognitive domains must be assessed in addition to memory), and which tests are more adequate for distinct populations, taking into account age-dependent, educational and cultural sources of bias [27]. Therefore, different assessment protocols to determine the degree of cognitive impairment may result in varying estimates of the cognitive deficits: more stringent tests are more sensitive to detect mild impairment of memory and other cognitive functions, whereas comprehensive batteries (for example, formal neuropsychological assessment) will more likely identify impairments in other cognitive functions beyond memory, favoring the identification of non-memory deficits and the diagnosis of multiple-domain MCI. As opposed to that, brief (function-oriented) cognitive batteries and screening tests may focus on the assessment of memory and overlook other cognitive domains. Thus, the lack of methodological uniformity to ascertain the degree and type of cognitive impairment across studies explains in part the discrepancies in prevalence and conversion rates [15].

**Figure 3:**
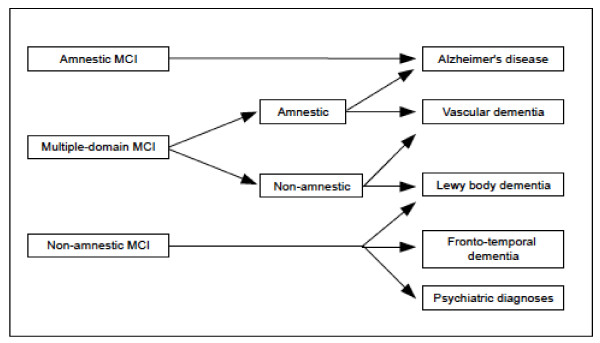
**Hypothetical outcomes according to distinct mild cognitive impairment (MCI) subtypes **[14,23].

The prognostic value of the MCI subtypes is an important issue on debate. The early definition of amnestic MCI supported the notion that the patients would present at the early stages of AD with signs of episodic memory impairment and progress linearly to a full-blown dementia syndrome. A similar assumption was attributed to other MCI subtypes and respective (theoretical) outcomes [28] (Figure 3). Nevertheless, epidemiological and clinical studies have questioned the association between MCI subtypes and specific dementia outcomes [29,30]. Individuals initially diagnosed as with MCI may show a long-term stability of cognitive deficits or even return to normal standards over time [31-33]. In fact, a substantial proportion of such patients may be reclassified as cognitively normal in a future evaluation. These cases are usually reported as 'unstable MCI'. It is still to be defined whether the first diagnosis was a false-positive artifact of cognitive testing, or if these individuals do recover normal cognitive function after having transient, subtle impairment. As it is, diagnostic instability is found in 5% to 20% of longitudinal samples of MCI [33]. These estimates tend to be inversely correlated with the level of certainty of raters on the clinical relevance of deficits at baseline.

Early studies assumed a linear trend between healthy cognition to MCI and dementia in older adults. These notions were based on analytical approaches that used time to event or last observation carried forward. Despite useful to determine conversion rates, these studies were not informative of the pattern of transitions between different clinical states. A different analytical strategy based on the Markov Chain model addressed the transitions between intact cognition, dementia and death in a subset of the Nun Study [34]. This model defines absorbing and non-absorbing states, which respectively represent the irreversible diagnoses of dementia (AD) or death, and the possibly reversible (or transitional) states of MCI. In this perspective, a plausible pattern of transitions between normal cognitive function, MCI and the diagnosis of AD could be: cognitively healthy subjects first develop single-domain amnestic deficits (incident MCI); upon follow-up, these subjects may retain this diagnosis (in spite of the possible exacerbation of memory impairment), or eventually develop deficits in other cognitive domains in addition to memory (usually attention and/or executive dysfunction). In this case, the diagnosis of single-domain amnestic MCI is updated to multiple-domain amnestic MCI. These patients may partially recover and return to the previous classification, but most commonly they retain the multiple-domain MCI status until the progression of memory and non-memory (mostly dysexecutive) deficits triggers functional impairment. At this point the clinical picture becomes compatible with mild dementia, and the diagnosis of dementia of the AD type is warranted. Reversal of deficits from this point becomes highly unlikely, which characterizes the absorbing state of dementia (AD) [20].

Therefore, the characterization of functional impairment is critical to establish the threshold between MCI and incipient dementia. Clinical and epidemiological studies have shown that patients with MCI may present subtle impairment in complex, instrumental activities of daily living (IAVDs), albeit not sufficient to impair independent living [35-38]. This is acceptable for the diagnosis of MCI according to the Mayo Clinic criteria [14]. Studies have suggested that the aggravation of functional deficits may occur independently of the worsening of memory impairment [39]. However, the magnitude of instrumental deficits to characterize conversion to dementia, given the prior diagnosis of MCI, has not been objectively defined, and this diagnosis depends primarily on clinical judgment.

This is important because the characterization of mild deficits in IAVDs in patients with MCI may hold a prognostic significance (that is, a higher risk to progress to AD/dementia). The objective evaluation of functionality has so far been neglected in the diagnostic investigation. It normally relies on the subjective report of patients and caregivers, or on the administration of functional scales to caregivers. There are several sources of bias in this form of assessment, namely the cognitive state of caregivers, the pattern of relationship between the patient and caregiver, their mood state and personality characteristics [40,41]. Thus, there is an urgent need for a better definition of functional impairment and for the operationalization of this assessment. In a recent study conducted in our group, the objective assessment of functional state provided evidence that patients with MCI may have mild but significant impairment in higher-order activities of daily living, such as shopping skills and managing finances, as compared to healthy older controls [42]. Functional deficits in patients with MCI and AD display a high and significant correlation with the performance on executive functions [21], and seem to be independent of age and formal schooling [43]. The magnitude of functional impairment in patients with MCI is similar among converters and non-converters, supporting the notion that mild functional impairment in also a feature of non-demented patients; however, a significant correlation between functional impairment and concentrations of phosphorylated Tau was found in the CSF of MCI patients who progressed to dementia [42], indicating that objective measures of IAVDs deficits is also correlated with well defined predictors of conversion.

As reviewed above, the concept of MCI may be sensitive to identify subjects that may develop AD/dementia, since most, if not all, individuals with predementia AD will present, at some point of the progression curve, with a long period of mild cognitive deficits prior to the onset of dementia. Nevertheless, as currently conceived, the clinically oriented diagnostic criteria for MCI yield a heterogeneous group of patients with distinct short-term and long-term outcomes. In other words, the specificity and the predictive value of the MCI diagnosis are low, and the cross-sectional identification of cases of prodromal AD may not reach adequate diagnostic accuracy if based solely on clinical tools [15]. Rather, it benefits substantially from the combination of clinical and biological information. In the next sections we will revise the recent developments on biochemical and neuroimaging biomarkers for the early diagnosis of AD. Of course, if such tests are unavailable, which may be the case in most healthcare settings, particularly in less favored countries, the expert interpretation of test results, the criterious observation of longitudinal measures, including the careful judgment of all available variables, is certainly the best alternative to drive clinical decisions.

## The search for biological biomarkers of Alzheimer's disease

The development of biomarker research in AD is a good example of the successful effort to translate the knowledge of key pathophysiological mechanisms of the disease into clinical applications. A biomarker is a characteristic that can be measured and evaluated as an indicator of the pathogenetic processes, or to ascertain the effect of pharmacological interventions on predefined biological cascades [44]. The ideal diagnostic marker for AD should meet at least three basic requirements: (i) reflect core neurobiological changes that characterize the disease process; (ii) be validated by post mortem studies, assuming that the neuropathological findings are gold standards of abnormalities affecting the same cascade; and (iii) be measurable as early as possible in the disease continuum, ideally at presymptomatic stages. Additional requirements include being non-invasive and simple to perform, precise and reliable, and adequate for large-scale screenings. Among many candidate markers, none has so far achieved universal acceptance, nor fully met the abovementioned criteria. Nonetheless, there has been significant progress toward this goal in the areas of CSF and neuroimaging biomarker identification, with attention focusing on the prediction of AD in the prodromal stages of disease and in high-risk groups.

## CSF biomarkers

The CSF may be considered an ideal source for viable biomarkers in AD. It is in intimate contact with the cerebral tissue, and pathological changes in the brain are often reflected in the CSF [45]. Among several potential diagnostic biomarkers, the most consistent findings have been obtained with the measurement of CSF concentrations of Aβ peptide (Aβ_42_), total Tau (T-Tau) and phosphorylated Tau (P-Tau). AD patients characteristically display low concentrations of Aβ_42 _and high concentrations of T-Tau and P-Tau. This pattern of CSF biomarkers is commonly referred to as the 'AD signature' in the CSF. The aforementioned biomarkers reflect core pathophysiological features of the disease [46], and have been validated in post mortem studies [47-49]. Increased concentration of T-Tau may be a less specific marker of axonal damage, as it can be found in vascular and other neurodegenerative dementias in addition to AD (for example, prion diseases). However, it bears a positive correlation with the speed and the magnitude of the neurodegenerative process. Decreased Aβ_42 _and increased P-Tau are more specific to AD. Aβ_42 _is a byproduct of the abnormal processing of the amyloid precursor protein (APP) leading to amyloidogenesis and formation of neuritic plaques. In addition, decreased concentrations of Aβ_42 _likely reflect its deposition in plaques, preventing its clearance through the CSF P-Tau illustrates the cytoskeletal changes that arise from the deregulation of microtubule homeostasis and ultimately cause axonal dysfunction and neuronal death. This marker is more specifically associated with AD, given the central role of Tau hyperphosphorylation in the formation of paired helical filaments (PHFs) and neurofibrillary tangles [50].

To date, over 100 studies have been published to support the notion that this AD-positive CSF pattern has good diagnostic accuracy to distinguish between normal ageing and AD (> 85%) and a positive predictive value (> 90%) to determine the dementia outcome in patients with MCI [51]. However, in the differential diagnosis of established dementia syndromes, the sensitivity/specificity profile to differentiate AD from other dementias is significantly lower [52]. Large-scale longitudinal studies of MCI cohorts consistently demonstrated that the presence of the 'AD signature' in the CSF has a good diagnostic accuracy (that is, >80%) discriminating patients with MCI who progress to AD ('MCI converters') from those who remain cognitively stable ('MCI-stable' patients) and healthy controls [51], as well as those MCI patients who progress to non-AD dementias [53]. These sets of data have been extensively replicated by different research groups worldwide [54-57]. Findings are largely confirmatory, as reinforced by a recent meta-analysis [58]. Taken together, these studies provide compelling evidence that the 'AD signature' in the CSF is a strong predictor of dementia outcome. MCI patients who convert to AD have a CSF biomarker pattern indistinguishable to that found in patients with dementia of the AD type; and MCI patients with progressive deficits (albeit not severe enough to characterize conversion) have a similar pattern to the former patients. Conversely, MCI patients with unstable (transient) MCI and those who display non-progressive deficits over time have a CSF biomarker pattern very similar to that found in healthy older adults.

However, a few methodological limitations need to be overcome before this knowledge can be translated into practical clinical practice. Although the determinations of CSF concentrations of these biomarkers using enzyme-linked immunosorbent assay (ELISA) or multiplex techniques (for example, xMAP; Luminex, Austin, TX, USA) have low coefficients of intralaboratory variability (5% to 10%), the high interlaboratory variation (20% to 30%) is a major obstacle for the comparison of data generated in different settings. Multiple sources of bias include preassay conditions (that is, lumbar puncture protocol, sample handling and aliquot storing prior to experimentation), intra-assay conditions (different methods and protocols for the determination of the concentrations of biomarkers), and post assay variations (for example, definition of norms for patients and controls to guide the interpretation of results) [59]. This situation is a major limitation for the establishment of multicentric cooperation. The establishment of gold-standard protocols to be shared by distinct laboratories [60] and the recent launch of a multicentric quality control program with over 40 laboratories around the world will hopefully overcome these limitations in the near future.

New technologies targeting Aβ oligomers in the CSF will add important insights in this field in forthcoming years. The neuropathology of AD has been linked to the accumulation of non-fibrillar forms of neurotoxic Aβ oligomers. There is evidence that soluble Aβ oligomers, more than amyloid fibrils *per se*, play a critical role triggering early pathological events of the amyloid cascade. High levels of Aβ oligomers are observed in the brain and in the CSF of patients with AD, underlining their potential for the early diagnosis of the disease [61]. In a recent study using a specific method for the detection of high molecular weight (40-200 kDa) Aβ species in the CSF, Fukumoto *et al*. [62] showed that the measurement of Aβ oligomers might be more accurate differentiating patients with MCI and AD from normal controls, as compared to the usual methods based on fibrillar forms of the peptide. Oligomerization partially explains the lowering of Aβ_42 _in the CSF of patients with AD, since the presence of Aβ oligomers can interfere with the analyses of the peptide by conventional methods, causing underestimation of Aβ levels due to epitope masking [63]. Therefore, the determination of Aβ oligomers in the CSF, in addition to being useful as a diagnostic marker for AD, can be also viewed as a potential surrogate marker for disease severity [62].

## Structural and functional neuroimaging

The substantial development of neuroimaging technologies in the last decade has contributed decisively to the search for non-invasive methods to ascertain the pathological changes that evolve in the AD brain. These advances result from new protocols for the analysis of structural imaging (such as volumetric assessments of regions of interest and voxel-based morphometry based on statistical maps) [64] and functional imaging with PET, addressing the metabolic changes that presumably antedate structural damage. More recently, the investigation of AD-specific biomarkers has been made possible with PET tracers that allow the *in vivo *intracerebral imaging of amyloid and Tau.

Structural changes in the brain in AD are mostly represented by global cerebral volumetric reduction, increased ventricular volumes and regional atrophy in structures of the medial temporal lobe (hippocampal formation and enthorinal cortex) [65]. Topographic gray matter loss correlates with Braak stages and may already be present in patients with very mild AD; such findings parallel the early cognitive symptoms found in the predementia phase of AD [8]. In comparison to AD, patients with MCI show a relative preservation of cerebral structures; however, these patients may have mild, but significant, volumetric changes and decreased cortical thickness in specific brain regions [65-67]. Increased grey matter loss is found in converters as compared to stable MCI subjects; these patients display volumetric reductions in hippocampal and parahippocampal structures and, to a lesser extent, in the posterior cingulate cortex, middle and inferior temporal gyri, fusiform gyrus, posterior cingulate gyrus, precuneus, temporoparietal junction, and frontal cortex [68-73]. A recent meta-analysis study indicated smaller left hippocampal volumes in converter versus stable MCI patients [74].

With respect to functional neuroimaging, the main metabolic changes observed in AD are global reductions in cerebral metabolism and perfusion as shown by FDG-PET and SPECT scans. These changes are observed in the temporoparietal junction, temporal, parietal and frontal lobes, hippocampal formation and posterior cingulate cortex [75,76]. As is the case for most methods of structural neuroimaging, patients with MCI show a pattern of changes that is intermediate between healthy older people and patients with AD [77,78]. Likewise, in prospective studies, MCI converters show a pattern of cerebral hypometabolism that is largely similar to that found in patients with mild AD, in particular in the posterior cingulate cortex and the hippocampal regions [79-83].

The development of new technologies to visualize and quantitate Aβ and Tau deposits *in vivo *within the brain is undoubtedly a major achievement in the field AD biomarker research. The first compound to be developed for human experimentation was the 'Pittsburgh Compound B' (PiB) [84], which is an ^11^C-labelled compound that binds intracerebral Aβ in mature amyloid plaques [85]. Other compounds are the amyloid-affinity compound ^18^F-BAY94-9172 [86], and the dual amyloid and Tau-binding compound 2-(1-{6-[(2-[18F]fluoroethyl)(methyl)amino]-2-naphthyl}ethylidene)malononitrile (FDDNP), which has the additional property of mapping neurofibrillary tangles in addition amyloid plaques [87].

In AD, there is an increased global cortical and regional retention of PiB and other compounds, particularly in the cingulate, temporal, parietal and frontal cortices [88]. Studies with amyloid imaging in mild AD have a very high sensitivity (over 90%), but the specificity is age dependent, due to the increasing deposition of Aβ over time in healthy older people. Important studies have shown negative correlations between intracerebral amyloid content (as shown by PiB scans) and CSF concentrations of Aβ_42 _in patients with mild AD as compared to controls [89,90].

As observed in other neuroimaging modalities, the PiB retention rates are also increased in patients with amnestic MCI, albeit less than in AD patients. Positive PiB scans predict conversion, and PiB retention (global and regional) correlates with cognitive performance [91,92]. In a prospective study, PiB-positive MCI patients had a higher conversion rate than PiB-negative patients; in addition, the amyloid load was negatively associated with time to conversion [93]. PiB retention was also observed in older subjects without cognitive complaints or dementia; it is noteworthy that a higher retention at baseline was associated with a worse cognitive performance and predicted a faster decline [94-97]. These findings are largely compatible with the CSF biomarkers as predictors of cognitive deterioration in non-demented older adults [98]. Finally, the combination of functional and structural imaging data reinforces the notion that the accumulation of Aβ in the AD brain precedes the onset of functional and structural changes (that is, high PiB retention correlates with the AD signature in the CSF and may be detected in the absence of significant brain atrophy [99-104]).

## Summary and future directions

A well defined pattern of CSF and imaging biomarkers can be characterized in AD. These biomarkers reflect core pathological changes that evolve in the prodromal phase of AD, including the predementia, and presumably the presymptomatic, stages of the disease. AD-related biomarkers identify with good accuracy non-demented patients with mild cognitive dysfunction who will ultimately progress to dementia, differentiating converters from healthy individuals and subjects with stable, non-progressive cognitive deficits. In addition, AD biomarkers may help to discriminate, although with lower accuracy, slow from rapid converting cases of MCI. The main biomarkers under investigation and their relationship with the pathological process in AD can be grossly subdivided into two main categories: (i) those reflecting core neuropathological changes of AD at the molecular level (for example, CSF biomarkers and amyloid imaging with PET), and (ii) downstream biomarkers reflecting secondary changes to brain structure and function, namely volumetric and metabolic changes to temporomedial structures (Table 1).

**Table 1:** Biomarkers under investigation for Alzheimer's disease

*Correlates:*	*Method/source:*	*Alzheimer's disease-related biomarkers:*
Molecularcore neuropathology	Cerebrospinal fluid	- Concentrations of amyloid-β_42_;
		- Total Tau and phosphorylated Tau;
	
	In vivo molecular imaging	- Intracerebral beta-amyloid load (e.g., PiB-PET, ^18^F-BAY94-9172);
		- Intracerebral aggregates of amyloid and tangle Tau(e.g., ^18^F-FDDNP);

Downstreamsecondary changes	Structural neuroimaging(MRI)	- Regional (medial temporal) atrophy (MRI)
		- Volumetry of hippocampus/entorhinal cortex (MRI)
		- Rate of brain/regional atrophy (MRI)
		- Voxel-based morphometry (VBM)^a^
		- Diffusion tensor imaging (DTI)^a^
	
	Functional neuroimaging(PET, SPECT, fMRI)	- Metabolic changes (FDG-PET)
		- Regional perfusion (SPECT)
		- Functional MRI^a ^and MRI perfusion-Functional connectivity^a^
	
	Neurochemistry	- Proton spectroscopy (^+^H-MRS)^a^

Associatedhomeostatic changes	Peripheral fluids(serum, plasma, platelets)	- Inflammatory markers (interleukins, cytokines)^a^
		- Oxidative stress (isoprostanes)^a^
		- Aβ40/Aβ42 ratio*;
		APP ratio^a^
		- Glycogen synthase kinase-3β activity^a^
		- Other markers of synaptic damage/neurodegeneration^a^

^a ^Less validated biomarkers; Aβ, amyloid-beta peptide; APP, amyloid precursor protein; MRI, magnetic resonance imaging; MRS, magnetic resonance spectroscopy; PET, positron emission tomography; SPECT, single-photon emission tomography; FDG, fluoro-deoxyglucose; PiB, Pittsburgh Compound B; FDDNP, 2-(1-{6-[(2-[18F]Fluoroethyl)(methyl)amino]-2-naphthyl}ethylidene)malononitrile.

In addition to providing biological support to the clinical diagnosis of AD, the establishment of biomarker technology has also favored the development of other important areas of research. First, the use of biomarker information can add important benefits to intervention trials, particularly with pharmaceutical compounds with disease-modifying properties. AD-positive biomarkers can be regarded as stringent inclusion criteria, defining more homogeneous samples of patients and therefore increasing the probability of success of randomized clinical trials; furthermore, the longitudinal reassessment of biomarkers can be viewed as a way to monitor specific biological outcomes of interventions with antidementia drugs, or to define proof-of-concept mechanisms of action of candidate drugs.

Secondly, given the long preclinical phase of AD, another potential use of biomarkers is the characterization of early signs of the disease in presymptomatic stages of the process. Evidence from epidemiological and autopsy studies support the hypothesis that there is a temporal lag of approximately a decade between significant accumulation of amyloid in the brain and the clinical onset of dementia. The percentage of amyloid-positive normal individuals detected at one given age closely parallels the percentage of individuals diagnosed with AD dementia a decade later [13]. In a longitudinal study with healthy older adults, changes in CSF biomarker levels associated with AD correlated with decline in cognitive functions, suggesting that these biomarkers may help identify early neurodegenerative processes of AD [105,106]. These notions have oriented recent task forces to develop diagnostic criteria for preclinical AD [107].

Finally, the actual prevention of dementia will be a tangible goal when the aforementioned challenges have been accomplished. In other words, the identification of individuals at high risk for developing dementia (including cognitively normal individuals at the presymptomatic stage of AD) and the effective treatment with pharmaceutical compounds with disease modifying properties will ultimately preclude (or at least attenuate) the subsequent neurodegeneration and eventual cognitive decline (Table 2).

**Table 2:** Putative clinical and biological markers of the distinct stages in the AD continuum (from normal cognition to dementia), and respective therapeutic interventions (clinically supported therapies and potential interventions with candidate drugs/strategies that still require experimental validation)

*Clinical stage*	*Underlying pathological mechanisms*	*Putative clinical and biological markers*	*Potential therapeutic interventions*
Asymptomatic (pre-clinical AD)	Intracerebral accumulation of amyloid-β	- CSF concentrations of Aβ_42_	- Cognitive reserve (education and level of intellectual functioning)
		- Molecular imaging (PiB-PET)	- Lifestyle changes (nutrition, physical fitness, reduction of stress)
		- Autossomal dominant mutation(APP, PS1, PS2 genes)	- Management of underlying factors (cardiovascular risk factors, toxic and comorbid conditions)
	
Prodromal (pre-dementia AD)	Aβ-related pathology(amyloid cascade)	- Episodic memory impairment(amnestic MCI)	- Anti-amyloid therapy: * immunotherapy anti-Aβ * modulation of β- and γ-secretase * anti-fibrillization agents and chelators
		- CSF concentrations of Aβ_42_	
		- Molecular imaging (PiB-PET)	
		- Autossomal dominant mutation(APP, PS1, PS2 genes)	
	
	Tau-related pathology (neurodegeneration)	- Multiple-domain amnestic MCI	- All above
		- CSF concentrations of Tau(total and phosphorylated Tau)	- Neuroprotective approaches(antioxidants, anti-inflammatory drugs)
		- Brain metabolism (FDG-PET)	- Tau-related therapies(GSK inhibitors, lithium)
		- Medial temporal lobe atrophy(volumetric MRI, VBM)	- Neurorestorative approaches(NGF, BDNF, stem cells)

Clinical dementia	Neuritic plaquesNeurofibrillary tangles	- Neuropsychological tests	- Antidementia drugs (cholinesterase inhibitors, memantine)
		- Functional assessment	- Cognitive training
		- Structural imaging (CT/MRI)	- Functional rehabilitation (ADLs)
		- Neuropathology	- Psychoeducation (caregivers)

AD, Alzheimer's disease; MCI, mild cognitive impairment; Aβ, amyloid-beta peptide; CSF, cerebrospinal fluid; APP, amyloid precursor protein; PS, pre-senilins 1 and 2; PET, positron emission tomography; PiB, Pittsburgh compound B; FDG, fluorodeoxyglucose; CT, computerized tomography scan; MRI, magnetic resonance imaging; VBM, voxel-based morphometry.

## Conclusions

Individuals with mild cognitive deficits do display signs of AD pathology, since approximately 50% are already in Braak neurofibrillary stage III or higher, and 20% are likely to be in more advanced stages of neuropathology [108]. It is likely that those with considerable brain or cognitive reserve will be able to compensate cognitive deficits until very close to the onset of the dementia, rendering the diagnostic investigation of predementia AD based solely on cognitive measures insensitive. Therefore, the development of biomarkers for AD is needed to target the severity of underlying brain pathology independently of brain reserve.

The measurement of AD-related biomarkers in the CSF or by neuroimaging methods improves diagnostic accuracy and predictive value of the clinical classification of patients according to the definition of MCI. The characterization of early clinical signs of AD (compatible with episodic memory impairment) with the support of one or more well established biomarkers has been recently proposed as the core feature required for the diagnosis of AD in the predementia stages [109]. This supports the clinical use of definition of MCI in the search for cases of prodromal AD [110]. The accurate identification of subjects with underlying AD pathology is an acute requirement for future trials with disease-modifying drugs [111,112]. However, as outlined in this review, there are critical methodological problems that still need to be overcome in order to enable the translation of this robust experimental knowledge into clinical practice.

In spite of the relevant contribution of clinical and biomarker research in the early diagnosis of AD, or even the characterization of the disease in asymptomatic or minimally symptomatic individuals, the use of these technologies raises the possibility of misidentification of cases. The incorrect classification of individuals as being at high risk for AD may lead to undue alarm and concern, in addition to unnecessary interventions. Therefore, before biomarker profiles are used in the general population, high specificity should be demonstrated in multiple populations. For the time being, the careful and comprehensive clinical judgment is mandatory to guide therapeutic decisions, even though the diagnostic hypothesis may be strongly reinforced by a positive biomarker. In the prospect that safe and effective, experimentally validated disease-modifying therapies become available in the near future, the reliable early detection of AD in the general population will become an essential tool in the prevention of this illness [113,114].

## Competing interests

The authors declare that they have no competing interests.

## Authors' contributions

The authors contributed equally to the selection and discussion of the literature reviewed in this work. The authors participated equally in the conception and preparation of the final manuscript.

## Pre-publication history

The pre-publication history for this paper can be accessed here:

http://www.biomedcentral.com/1741-7015/8/89/prepub
